# Combined central and peripheral demyelination: A case report and literature review

**Published:** 2019-01-05

**Authors:** Hamza Nouha, Hdiji Olfa, Farhat Nouha, Feki Sawsan, Sakka Salma, Haj Kacem Hanen, Dammak Mariem, Mhiri Chokri

**Affiliations:** 1 Department of Neurology, Habib Bourguiba Hospital, Sfax, Tunisia; 2 Department of Immunology, Habib Bourguiba Hospital, Sfax, Tunisia

**Keywords:** Demyelinating Diseases, Multiple Sclerosis, Chronic Inflammatory Demyelinating Polyradiculoneuropathy, Plasma Exchange, Neurofascin

There was a 20-year-old man who presented in May 2017 with an acute static and kinetic cerebellar syndrome. Cerebral magnetic resonance imaging (MRI) showed T2 and flair subcortical and periventricular hyperintense lesions, one of them was pseudotumor-like with peripheral contrast enhancement (Figure 1). Lumbar puncture (LP) revealed 10 white blood cells (WBC), proteinorrhachia of 4.16 g/l, glycorrhachia of 3.72 mmol/l, normal immunoglobulin G (IgG) index (IgG index: 0.49), and no oligoclonal bands. Visual evoked potentials showed bilateral axonal-demyelinating optical neuritis. The diagnosis of multiple sclerosis (MS) was retained after excluding other inflammatory diseases (normal immunological assessment, no bipolar aphthous, neither skin lesions nor ocular damage on ophthalmic examination). The patient received 3 g of methylprednisolone (1 g/day), and was put on interferon β-1a (Avonex®). The evolution was towards the appearance of a new relapse in June 2017. He received 5 g of methylprednisolone during 5 days with initially mild improvement. In August 2017, the patient reconsulted for progressive weakness of the four limbs. Our patient was bedridden, and the neurological examination found flaccid tetraplegia with important amyotrophy. The second MRI showed the same aspect of lesions but without gadolinium enhancement, and the nerve conduction study [electromyography (EMG) and nerve conduction velocity (NCV)] showed severe sensory-motor demyelinating neuropathy (Table 1). Control LP revealed an increase in protein level at 6.37 g/l and 2 Wight elements with no oligoclonal bands.

**Figure 1:**
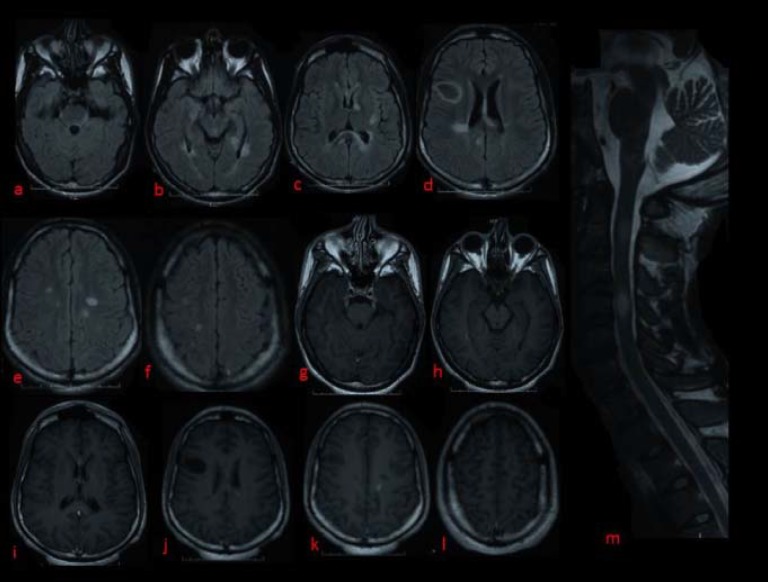
Magnetic resonance imaging (MRI) findings prior to therapy. Axial sections of cerebral MRI FLAIR show hyperintense periventricular and subcortical lesions and a tumor-like frontal lesion with peripheral open ring gadolinium enhancement (g, h, I, j, k, l), axial sections of cerebral MRI in post-contrast T1 sequences show 3 actives lesions with contrast enhancement (a, b, c, d, e, and f), and longitudinal section of the spinal cord in T2 sequence show 2 cervical hyperintense lesions (m).

The diagnosis of chronic inflammatory demyelinating polyradiculoneuropathy (CIDP) was made referred to European Federation of Neurological Societies/Peripheral Nerve Society (EFNS/PNS) criteria 2010. Faced to this association of central and peripheral demyelinating lesions, we continued with etiological investigations that were negative for human immunodeficiency virus (HIV), hepatitis B and C serology, antineutrophil cytoplasmic antibody (ANCA), and anti-nuclear antibody (ANA), thyroid hormones, vitamin B12, Protein immune-electrophoresis in sera (IEP), calcium, lactate/pyruvate ratio, and herpes viruses [herpes simplex virus (HSV), Epstein-Barr virus (EBV), and varicella zoster virus (VZV)] serology in favor of an old immunization. Antibodies directed against paranodal proteins including anti-contactin-1, anti-contactin-associated protein 1 (CASPR-1), anti-neurofascin-155, and anti-neurofascin-186 antibodies were negative. Methylprednisolone pulse therapy for five times was ineffective.

**Table 1:** Summary of electromyography (EMG) and nerve conduction velocity (NCV) studies

**Tests**	**Normal value**	**First hospitalization**		**Last hospitalization**	
		**Right**	**Left**	**Right**	**Left**
Motor nerve conduction					
DML (ms)					
Median	≤ 3.8	3.8	3.9	0	0
Ulnar	≤ 3.2	3.2	2.4	0	0
Peroneal	≤ 5	4.6	4.4	0	0
tibial	≤ 5.5	3.7	4.5	0	0
F waves (Median)	25-30	29.2	30.4		
F waves (Tibial)	45-50	46.5	58.1		
CMAP (mV)					
Median	≥ 6	11.2	5.8	0	0
Ulnar	≥6	9.2	8.6	0	0
Peroneal	≥ 3	2.7	2.6	0	0
Tibial	≥ 6	2.9	3.0	0	0
MCV (m/s)					
Median	≥ 45	50.0	47.0		
Ulnar	≥ 45	58.5	53.8		
Peroneal	≥ 42	42.9	45.8		
Tibial	≥ 42	0	0		
Sensory nerve conduction					
SNAP (mV)					
Ulnar	≥ 10	9.2	12.0	0	0
Median	≥ 15	49.0	58.0	ND	ND
Musculocutaneous	≥ 10	13.0	11.0	1.9	1.4
Sural	≥ 10	8.2	6.4	3.5	1.3
SCV (m/s)					
Ulnar	≥ 45	65.2	65.9		
Median	≥ 45	55.6	69.8	ND	ND
Musculocutaneous	≥ 40	52.4	50.0	28.1	26.2
Sural	≥ 40	41.4	40.5	19.2	2..8

DML: Distal motor latency; CMAP: Compound muscle action potential; MCV: Motor nerve conduction velocity; SNAP: Sensory nerve action potential; ND: Not done; SCV: Sensory conduction velocity

The Avonex® was stopped, and the patient underwent 5 sessions of plasma exchange and re-education with a mild improvement. In fact, he could ambulate with double aid; the amyotrophy had remarkably regressed with present reflex in upper limbs. For the background treatment, we decided to put the patient under Endoxan with stabilization.

Combined central and peripheral demyelination (CCPD) is a large term that was proposed to describe a situation associated with demyelinating lesions of the central and peripheral systems (CNS and PNS). The combination of CIDP and CNS involvement similar to MS represent a particular type of CCPD. In our patient, peripheral signs were dominating, at the last relapse, and the diagnosis of CIDP associated to MS was made. It was systematic to think to a simple coincidence of the two diseases. But, the low prevalence of MS and the scarcity of CIDP, as well as the presence of atypical features, prompted us to find an unusual and rare condition^1^ that united these two diseases. Recently, this association of CIDP and MS was increasingly recognized and reported in the literature as clinical cases which allowed to be better characterized. Recently an anti-neurofascin-155 antibody was found in several demyelinating diseases of the nervous system with low prevalence. This antibody likes to be more specific of the association of CIDP with MS when it was positive in 86% in the Japanese study of Kawamura, et al.^2^ It was also highlighted in clinical cases that reported this association.^3^ The presence of the neurofascin-155 in the CNS and PNS and the high prevalence of the anti-neurofascin-155 in CCPD suggested its important pathogenic role in this condition. Nevertheless, this antibody was sometimes absent during this disease, as it was in our patient and in others cases in the literature,^4^ what was proving that the physiopathological process is still poorly understood. This CCPD syndrome with so far unknown immunological target(s) allowed us to research for others antibody directed against others paranodal proteins which are shared between CNS and PNS. In fact, the role of contactin-1 and CASPR-1 was largely described in CIDP, but recently, evidence for multiple and critical functions of contactin-1 in central myelin was documented,^5^ and the implication of CASPR in central myelinisation is also known. Atypical signs characterized this demyelinating disease, which was often associated with marked hyperproteinorrhachia. In fact, the oligoclonal bands were often absent, the pseudo-tumor lesions were frequent, and the bilateral involvement of the optic nerves too. For our patient, there were a hyperproteinorrhachia at 6.36 g/l, a pseudo-tumor lesion on cerebral MRI, and a bilateral optic neuritis. The worsening of the symptomatology with corticosteroids is unusual in the CIDP whereas it was more frequent in the peripheral signs during the CCPD. There is no clear therapeutic consensus for this disease, but plasma exchange seems to have a beneficial effect on symptoms improvement.^3^ This was confirmed in our patient.

## Notes:

**How to cite this article:** Nouha H, Olfa H, Nouha F, Sawsan F, Salma S, Hanen HK, et al. Combined central and peripheral demyelination: A case report and literature review. Iran J Neurol 2019; 18(1): 35-7.

## References

[1] Zephir H, Stojkovic T, Latour P, Lacour A, de Seze J, Outteryck O (2008). Relapsing demyelinating disease affecting both the central and peripheral nervous systems. J Neurol Neurosurg Psychiatry.

[2] Kawamura N, Yamasaki R, Yonekawa T, Matsushita T, Kusunoki S, Nagayama S (2013). Anti-neurofascin antibody in patients with combined central and peripheral demyelination. Neurology.

[3] Shimizu M, Koda T, Nakatsuji Y, Ogata H, Kira JI, Mochizuki H (2017). A case of anti-neurofascin 155 antibody-positive combined central and peripheral demyelination successfully treated with plasma exchange. Rinsho Shinkeigaku.

[4] Garcia J, Tchikviladze M, Evrard S, Wang A, Lapergue B, Auliac S (2017). Anti-neurofascin 155 paranodopathy with central and peripheral nervous system involvement: A case and review of the literature. Pratique Neurologique - FMC.

[5] Colakoglu G, Bergstrom-Tyrberg U, Berglund EO, Ranscht B (2014). Contactin-1 regulates myelination and nodal/paranodal domain organization in the central nervous system. Proc Natl Acad Sci USA.

